# Mixed Culture of Yeast and Lactic Acid Bacteria for Low-Temperature Fermentation of Wheat Dough

**DOI:** 10.3390/molecules30010112

**Published:** 2024-12-30

**Authors:** Wiktoria Liszkowska, Ilona Motyl, Katarzyna Pielech-Przybylska, Urszula Dziekońska-Kubczak, Joanna Berłowska

**Affiliations:** 1 Department of Environmental Biotechnology, Lodz University of Technology, Wolczanska 171/173, 90-530 Lodz, Poland; ilona.motyl@p.lodz.pl (I.M.); joanna.berlowska@p.lodz.pl (J.B.); 2 Institute of Fermentation Technology and Microbiology, Lodz University of Technology, Wolczanska 171/173, 90-530 Lodz, Poland; katarzyna.pielech-przybylska@p.lodz.pl (K.P.-P.); urszula.dziekonska-kubczak@p.lodz.pl (U.D.-K.)

**Keywords:** low-temperature fermentation, volatile organic compounds, sourdough, yeast, lactic acid bacteria

## Abstract

There is growing interest in low-temperature food processing. In the baking industry, low-temperature fermentation improves the production of natural aroma compounds, which have a positive impact on the sensory profile of the final product. The aim of this study was to develop a yeast–lactic acid bacteria starter culture that effectively ferments wheat dough at a temperature of 15 °C. The microorganisms were selected based on their enzymatic activity and ability to grow at low temperature. The fermentation activity of the yeast and mixed cultures was assessed enzymatically. The biosynthesis of volatile organic compounds was quantified using the HS-GC-MS technique. Samples fermented by *S. cerevisiae* D3 were characterized by the highest concentration of volatile organic compounds, especially esters. The addition of lactic acid bacteria increased not only the biosynthesis of volatile organic compounds but also the productivity of carbon dioxide during dough fermentation. Based on both dough expansion and the profile of volatile organic compounds, a mixed culture of *S. cerevisiae* D3 and *L. brevis* B46 was selected as the most effective starter for low-temperature fermentation.

## 1. Introduction

Wheat flour is the most popular type of flour. It is also the main cereal crop consumed in Poland. Ten times more wheat flour is produced than rye flour. Wheat flour is the most commonly used raw material for bread production, due to its excellent physical and chemical properties [1]. Wheat flour has a high gluten content, which improves dough structure. Traditionally, wheat flour type 750 is used in bread making, and wheat flour type 550 is used for making rolls [2,3]. There are four wheat-flour-specific sugars, which are consumed by yeasts during fermentation: fructose, glucose, sucrose, and maltose. The amounts of these substrates change during the technological process, due to fermentation and the production and secretion of amylolytic enzymes [4]. The evolution of the sugar content of wheat flour and dough during fermentation is extremely important. Each type of wheat flour differs not only in its sugar content but also in its ash content, acidity, moisture, colour, and gluten content. The moisture content of high-quality flour can reach up to 15%. Higher water content contributes to the clumping of the flour and the development of microorganisms that reduce the quality of the end product [5,6]. Other key factors include the temperature and consistency of the baking process. These factors determine the growth of microorganisms during fermentation.

Yeasts are used as leavening agents and are responsible for producing key aroma and flavour compounds. Lactic acid bacteria produce organic acids, which have two important roles: they acidify the fermentation environment and limit the development of unwanted microorganisms, such as moulds. As consumer demand increases for healthier and higher quality products, sourdough breads are becoming increasingly important [7,8].

Traditionally, a five-phase process is employed for products derived from rye flour, whereas for products derived from wheat flour, two production methods are employed. The first approach is a single-phase method, which involves combining all the ingredients at once. This is the fastest and simplest method. The dough is ready for baking in about 2–3 h. The second, two-phase method involves preparing a sponge with the addition of yeast which is then combined with the remaining ingredients to make the dough. This method takes longer than the single-phase method (approximately 3–4 h). The temperature and the texture of the water–flour mixture and dough change during each of the phases. The sponge is produced at 26–27 °C in a more hydrated environment. This promotes rapid multiplication of the yeast. The dough is then fermented at 30 °C. The use of a two-phase process promotes the biosynthesis of aroma compounds, which improve the sensory properties of the bread and reduce the need for enhancers [9].

As baking technologies continue to develop, new possibilities have emerged to shorten the time between baking and consumption while maintaining the sensory values of the product [10]. Commercially used postponed baking technology allows producers to delay the baking time. The dough is initially prepared and then cooled to 0–5 °C or frozen. This inhibits the dynamic expansion of the dough by limiting the metabolic activity of the yeast. The baking of bread can be delayed by up to several hours. After this time, the dough is proofed at a higher temperature. The metabolic activity of the yeast increases, and the dough expands [11,12]. However, the reduced temperature does not completely stop the biosynthesis of aroma compounds, so the dough can acquire sensory values even before the actual fermentation process. Although the biosynthesis of aroma compounds takes longer at low temperatures, it offers numerous advantages. The low-temperature process has been demonstrated to result in a higher sensory evaluation of the baked goods. The reduced temperature of the process affects the production of natural aroma compounds, which have a beneficial effect on the sensory profile of the bread [11,13]. Scientific studies indicate that low temperatures favour the production of volatile organic compounds, which are responsible for the aroma of the final product [14,15]. They mainly derive from yeast metabolism and include compounds such as organic acids, for example, acetic acid, and esters, for example, ethyl acetate, ethyl hexanoate, and ethyl octanoate. Birch and coworkers examined the optimal temperature for fermentation and the synthesis of key aroma compounds [15]. They concluded that leavening dough at a lower temperature for a longer period increases the formation of aroma compounds. These results were obtained for dough fermented with yeast only. Xu and coworkers examined the efficacy of low-temperature sourdough fermentation using mixed cultures of yeast and lactic acid bacteria. The sensory profile of bread with the addition of sourdough fermented at 10 °C received better marks from a sensory panel [16]. Volatile organic compounds in bread are essential for consumer acceptance and product identification. The fermentation process, conducted by yeasts and lactic acid bacteria, determines the unique sensory profile of the final product.

Delayed baking also enables the reorganization of work, by eliminating the night shift and reducing the number of staff, significantly impacting the financial aspect of managing a bakery [17]. Low-temperature fermentation is already used in the wine and brewing industry, and other food industries have started to research low-temperature industrial processes [18]. However, despite the reduction in staff costs, postponed baking technology may lead to an increase in production costs compared with one- and two-phase methods. Refrigeration equipment can be a significant expenditure for small enterprises [19].

Traditional baking uses spontaneous fermentation. A more convenient alternative is to use a starter culture, which significantly reduces the time of the whole process. At a lower temperature, spontaneous fermentation is inhibited because the process temperature is not favourable for the development of microorganisms. Therefore, it is necessary to use a starter culture that is capable of rapidly controlling the environment. Mixed starter cultures are sought that show good metabolic activity during co-fermentation at low temperatures. This will reduce costs associated with incubating the dough at very low or higher temperatures, while improving the sensory profile of the final product.

Low temperature processes offer several technological and economic benefits for the baking industry. Firstly, they enable reducing electricity costs by eliminating the need to heat rooms and fermentation chambers. Due to the extended duration of the process, it is possible to reorganize work in bakery plants to avoid the need for night shifts. Low-temperature fermentation also results in a better sensory profile, while limiting the development of undesirable microflora responsible for food spoilage. This reduces the need to use artificial flavours and preservatives.

Taking into consideration the above, the aim of this study was to develop a mixed starter culture which effectively ferments wheat dough at a temperature of 15 °C. Both yeast and lactic acid bacteria have their optima above the target temperature. Thus, the first novelty of this research was the use of yeast and bacteria that not only operate at 15 °C but were adapted to the target temperature. This resulted in maintaining good metabolic activity at that temperature. Firstly, the most important parameters were measured, including yeast growth in the flour–water mixture at low temperature, basic carbohydrate content, enzymatic activity, the ability of the tested strains to produce carbon dioxide at a low temperature, and the biosynthesis of key aroma compounds. To improve the productivity of carbon dioxide and the formation of volatile organic compounds during fermentation, lactic acid bacteria were incorporated. In industry, lactic acid bacteria are not commonly used for the fermentation of wheat flour. These microorganisms are employed mainly to produce rye sourdough for mixed bakery products. Experiments were also conducted using wheat flour type 650, which had not previously been used in such processes. Thus, the second novelty of this research is the type of raw material used in the process of low-temperature fermentation.

## 2. Results and Discussion

Low-temperature processes offer several technological and economic benefits for the baking industry. Firstly, they enable reducing electricity costs by eliminating the need to heat rooms and fermentation chambers [20]. Due to the extended duration of the process, it is possible to reorganise work in bakery plants to avoid the need for night shifts.

In this case, some practical implications such as extended fermentation time or specific infrastructure requirements for low-temperature fermentation should be taken into consideration. The requirements can be met by plants of various sizes, taking into account the technological development of the bakery industry and new automated solutions for delayed baking.

Low-temperature fermentation also results in a better sensory profile, while limiting the development of undesirable microflora responsible for food spoilage. This reduces the need to use artificial flavours and preservatives. Such processes, however, require the specific screening of microorganisms taking into account their ability to grow and their biochemical properties. Conventional yeasts for this study were chosen due to their well-known properties, as well as their recognition as being safe for human consumption (GRAS status). These are also the microorganisms commonly used in the baking industry.

The yeasts were selected based on their growth rate at 15 °C, indicated as optical density and CFU/mL (Supplementary Materials: Figures S1 and S2), as well as based on their metabolic activity for assimilating sugars (Supplementary Materials: Table S1) and producing volatile organic compounds in the model medium (Supplementary Materials: Table S2). The summary of the strains selected for the experiments was presented in Table 6 in Section 3.

The activity of the yeast was evaluated by API ZYM (Table 1). All the tested yeasts showed good enzymatic activity at 15 °C. Enzymatic activity is closely related to the formation of aroma compounds [21]. Next, the sponges were quantitatively analysed for the biosynthesis of volatile organic compounds.

To improve the fermentation process, lactic acid bacteria strains were used. Regarding lactic acid bacteria, the strains were isolated from the environment that leads to enhanced resistance to changing cultivation conditions. The strains were selected based on their ability to grow at a temperature of 15 °C and their enzymatic activity (API ZYM). The summary of the strains selected for the experiments was presented in the Table 7 in Section 3. Adaptation to low temperature was accomplished through multiple passages of pure cultures. Three strains showing the best biomass growth (Supplementary Materials: Figure S3) and with confirmed enzymatic activity at low temperatures (15 °C) were selected (Table 2). The enzymatic profiles of the lactic acid bacteria included five more enzymes than were identified in the case of yeast. These abilities can be used during the co-fermentation of mixed cultures to release the carbon source for both microorganisms. The looser structures of complex polymers also make the final product more digestible for humans. Esterase (C4) and esterase lipase (C8) enzymes are involved in the synthesis and breakdown of esters, such as ethyl acetate and isoamyl acetate, which are characterised by fruity aromas. The activity of leucine, valine, and cystine arylamidases indicates the ability to metabolise amino acids, leading to the production of higher alcohols, such as isoamyl alcohol and 2-methyl-1-propanol, which are responsible for fusel and pungent odours. Acid phosphatase and Naphthol-AS-BI-phosphohydrolase are involved in metabolic pathways associated with phosphate transformations, which may indirectly influence the synthesis of alcohols and esters. α-glucosidase, particularly present in *S. cerevisiae* LOCK0153, enables the breakdown of complex sugars, increasing the availability of precursors such as glucose and fructose, which are converted into aldehydes and esters [22].

Moreover, in addition to the enzymatic activities identified in both yeasts and lactic acid bacteria, the distinct activity of ß-galactosidase and ß-glucosidase is characteristic of the bacterial strains. High activity of ß-galactosidase and ß-glucosidase in all strains (values of 5) indicates the ability to break down complex sugars, which may enhance the production of aromatic precursors such as aldehydes and alcohols [23].

### 2.1. Yeast Growth in Wheat Sponge at 15 °C

Due to their ability to adapt quickly to new conditions, yeasts are commonly and increasingly used in biotechnological processes. In particular, there is growing scientific interest in the adaptation of yeast to low temperatures. In recent years, there have been intensive efforts to diversify fermented products, focusing not only on using a variety of raw materials but also on optimizing existing production processes. In the study conducted by Liszkowska and coworkers, eight different environmental yeast strains, including *S. cerevisiae*, *K. barnettii*, *W. anomalus*, *H. uvarum*, and *P. fermentans* were characterized [24]. In the model medium, the stationary phase at 15 °C was reached even after 96 h. Therefore, in the sourdough environment, this time can be extended by one day to allow the accumulation of fermentation by-products, which are often synthesised after the stationary phase is reached.

**Figure 1:**
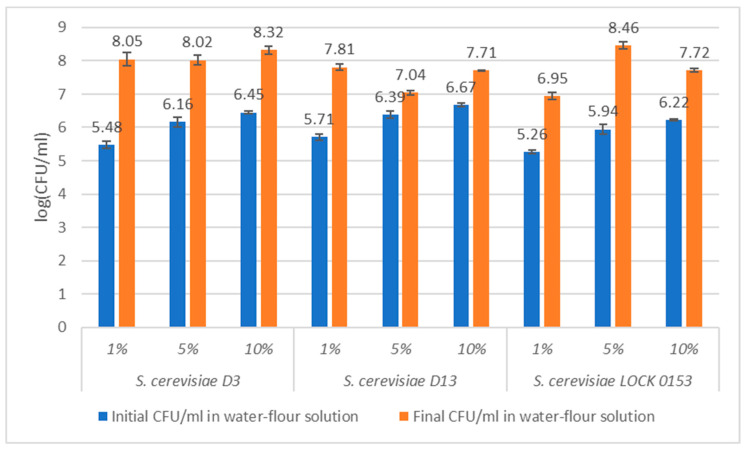
Concentration (CFU/mL) of the examined yeast strains before and after fermentation of the prepared water–flour suspension. Abbreviations: 1%, 5%, 10% refer to the inoculation doses in the samples.

The use of yeast in sourdoughs has attracted significant attention. However, only a few studies have focused on their use and activity in sponge [21,25,26,27]. In this study, the use of environmental *Saccharomyces* yeast strains that grow well at 15 °C for sourdough making and dough leavening was investigated. Wheat sourdoughs were fermented at 15 °C for 5 days. The viability of the applied yeasts was estimated by measuring the CFU/mL at the beginning and end of the process. To determine the most suitable concentration of yeast cells, three inoculation amounts were tested. The inoculation concentration is usually around 1–3%; however, these data are presented for inoculum with a high concentration of yeast cells [4,9,28,29,30,31]. Initial concentrations reported in the literature for sourdough are around 10^7^ CFU/mL [32]. In this study, the initial yeast concentration in the water–flour mixture after inoculation varied in the range of 10^5^–10^6^ CFU/mL. Given the yeast concentration and temperature of the process (which does not promote rapid growth of microorganisms), higher inoculum doses were chosen to differentiate the final concentration of yeast cells. The results are presented in Figure 1.

The inoculation size differentiated the final CFU/mL. *Saccharomyces cerevisiae* D3 showed the highest value in the sourdough with 10% inoculum and had the best concentration throughout almost the whole experiment. The other samples, with 1% and 5% inoculum, showed a slightly lower final biomass level. Yeast from the pure culture collection, *Saccharomyces cerevisiae* LOCK 0153, grew most efficiently in the sponge with 5% inoculum (exceeding 10^8^ CFU/mL). Strain *Saccharomyces cerevisiae* D13 presented the highest final concentration of cells in sourdough with 1% inoculum, but it did not exceed 10^8^ CFU/mL. Compared with the other strains, the increase in biomass production was lower. The results obtained in this study are similar to those reported in the literature on microbiota diversification. Komatsuzaki and coworkers investigated the biomass in sourdoughs at two different temperatures, 8 °C and 28 °C. The production of yeast in both cases exceeded 10^8^ CFU/mL [33]. Considering the lack of data on fermentation processes at low temperatures, it is possible to compare this study only partially with similar results. It is, however, promising that various yeast isolates selected for this experiment can grow well at 15 °C, based on the results of other research from processes conducted at an optimal temperature of around 30 °C [34].

#### 2.1.1. Changes in Sugar Content After Fermentation of Sponge

**Figure 2:**
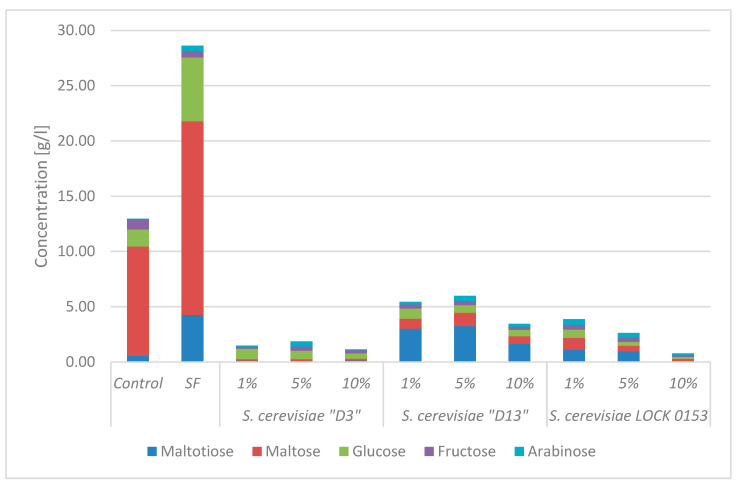
Sugar content in samples after fermentation. Abbreviations: “Control” indicates sample before fermentation; “SF” indicates spontaneous fermentation; 1%, 5%, 10% refer to the inoculation levels in the samples.

The selection of an appropriate yeast is crucial for ensuring effective fermentation, as its growth largely depends on the availability of easily digestible carbohydrates. The predominant sugars present in wheat flour are glucose polymers, including maltose and maltotriose, as well as fructose polymers, namely fructans and arabinoxylans. Their chains contain simple sugars that are easily assimilated by yeast. The origin of a particular strain may influence the assimilation ability of a particular yeast species. Therefore, the concentrations of five different forms of carbohydrates were determined in this study. The results are presented in Figure 2. A control sample composed of water and wheat flour was examined for the presence of selected sugars prior to fermentation. After controlled fermentation using three different yeast strains, the concentrations of sugars in the sponges were determined. To compare the changes occurring during the process, their concentrations were also measured in a spontaneously fermented sample.

Significant changes in the concentrations of the selected sugars were observed after fermentation compared with their initial concentrations. During spontaneous fermentation, an increase was observed in all analysed compounds, with the exception of fructose. However, after controlled fermentation with three yeast strains, a significant decrease in the concentrations of all carbohydrates was observed. Each strain had a slightly different carbon utilization profile. In samples containing *S. cerevisiae* D3, decreases in the concentrations of maltotriose, maltose, glucose, and fructose were observed. After the process, the sponge with 10% inoculum contained the lowest concentration of sugars. Arabinose, however, was not used. The concentration of this sugar increased during fermentation conducted with 5% inoculum of *S. cerevisiae* D3. The sugar concentrations in sponges fermented with the *S. cerevisiae* LOCK 0153 strain were similar to those in samples fermented with the *S. cerevisiae* D3 strain. The smallest differences were observed with 10% inoculum. Nevertheless, maltose was utilized to a much lower extent. The sugar utilization profile was different in samples with 1% and 5% inoculum compared to samples with 10% yeast addition. The concentrations of maltotriose and arabinose were higher, with a significant decrease in the concentrations of fructose, glucose, and maltose. *S. cerevisiae* D13 strain had a weaker ability to use maltotriose produced during the fermentation process. The concentration of this sugar was highest in the sample with the addition of 5% inoculum and was lowest in the sample with 10% yeast inoculum. A comparable utilization of glucose, maltose, and fructose was also observed. Comparing the obtained results with the studies conducted by Codină and coworkers, it was observed that, regardless of the process temperature, the yeast utilized the primary sugars—glucose, fructose, and maltose. However, lowering the temperature also resulted in the increased utilization of maltotriose [4].

#### 2.1.2. Biosynthesis of Volatile Organic Compounds in Wheat Sponge

**Figure 3:**
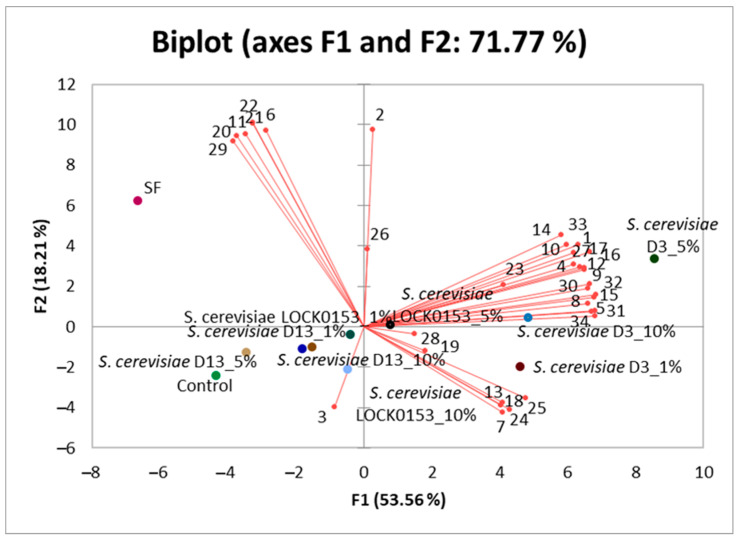
Principal component analysis (PCA) biplot for sponges. Variables: 1—Acetadehyde; 2—Acetone; 3—Ethyl formate; 4—Isobutyraldehyde; 5—1-Propanol; 6—2,3-Butanedione; 7—Ethyl acetate; 8—Isobutyl alcohol; 9—Isovaleraldehyde; 10—2-Methylbutyraldehyde; 11—1-Butanol; 12—Ethyl propionate; 13—Propyl acetate; 14—Acetaldehyde diethyl acetal; 15—3-Methylbutanol; 16—2-Methylbutanol; 17—Ethyl isobutyrate; 18—Isobutyl acetate; 19—Hexanal; 20—Ethyl butyrate; 21—Ethyl lactate; 22—Butyl acetate; 23—1-Hexanol; 24—3-Methylbutyl acetate; 25—2-Methylbutylacetate; 26—2-Pentylfuran; 27—Ethyl hexanoate; 28—Hexyl acetate; 29—Isoamyl butyrate; 30—Ethyl heptanoate; 31—2-Phenylethanol; 32—Ethyl octanoate; 33—Ethyl nonanoate; 34—Ethyl decanoate.

Inoculated samples of wheat sponges after fermentation were subjected to headspace (HS) analysis coupled with mass spectrometry (GC-MS) to quantitatively identify the presence of volatile organic compounds synthesized during the fermentation of sourdoughs at 15 °C with the addition of various amounts of tested yeast strains (detailed concentration of the volatile organic compounds are available in Supplementary Materials Tables S3–S5). During the chromatographic analysis, 34 volatile compounds were quantitively assessed. These compounds belong to the three main groups: carbonyl compounds, higher alcohols, and esters. To illustrate the differences in the qualitative and quantitative composition of volatile compounds in the tested sponge samples, the obtained data were subjected to principal component analysis (PCA). Two components were used, PC1 and PC2, which explained 53.56% and 18.21% of the total variance, respectively, collectively accounting for 71.77% of the information contained in the model. The first principal component divided the studied sponges into two groups (Figure 3).

The first group included all three sponges fermented by yeast strain *S. cerevisiae* D3, with the highest contribution of the observations (36.54%) attributed to the sponge sample with an inoculum amount of 5%. The second group comprised the remaining sponges, including the control and spontaneously fermented samples. The sample after spontaneous fermentation showed the highest contribution of the observations (22.27%) in this group. The other sponge samples did not exhibit clear separation, indicating that the qualitative and quantitative composition of the volatile compounds was similar between these samples.

PC1 was positively strongly correlated with most of the identified compounds, including aldehydes (acetaldehyde, isobutyraldehyde, isovaleraldehyde, and 2-methylbutyraldehyde), acetals (1,1-diethoxyethane), esters (ethyl hexanoate, ethyl heptanoate, ethyl octanoate, ethyl nonanoate, ethyl decanoate, ethyl isobutyrate, and ethyl propionate), as well as higher alcohols (2-phenylethanol, 2-methyl-1-butanol, 3-methyl-1-butanol, 2-methyl-1-propanol, and 1-propanol). The correlation coefficients with these compounds had high values, ranging from 0.826 to 0.973. Despite classifying *S. cerevisiae* D3 yeast-fermented sponges into one group, differences can be observed between them depending on the amount of inoculum added to the sponge. The sponge inoculated with 5% of *S. cerevisiae* D3 yeast had the highest concentrations of most of the identified compounds. On the other hand, the sample after spontaneous fermentation had the largest contribution (56.984%) to the second component (PC2). There are clear distinctions between the sample after spontaneous fermentation, the control sample (without fermentation), and the *S. cerevisiae* D13 (1%, 5%, and 10%) and *S. cerevisiae* LOCK 0153 (1%, 5%, and 10%) samples. The sample after spontaneous fermentation is distinguished by high concentrations of acetone, 1-butanol, 2,3-butanedione, isoamyl butyrate, ethyl butyrate, butyl acetate, and ethyl lactate. The correlation coefficients with these compounds ranged from 0.761 to 0.837.

Based on the results, it can be concluded that the fermented sponges after fermentation were characterized by different concentrations of volatile organic compounds, depending on the yeast strain as well as the inoculum amount.

### 2.2. Fermentation of Wheat Dough by Yeast and Lactic Acid Bacteria

#### 2.2.1. Carbon Dioxide Production During Yeast Fermentation

For the bakery industry, the ability of microorganisms to produce both organic and inorganic compounds is as important as their growth rate during fermentation. Carbohydrates present in the dough are utilized to produce carbon dioxide and ethanol through glycolysis and further pyruvate reduction. Although ethanol can have an impact on the properties of dough, the crucial compound is carbon dioxide. Carbon dioxide causes leavening by spreading throughout the entire dough, giving it a porous structure [35,36].

**Figure 4:**
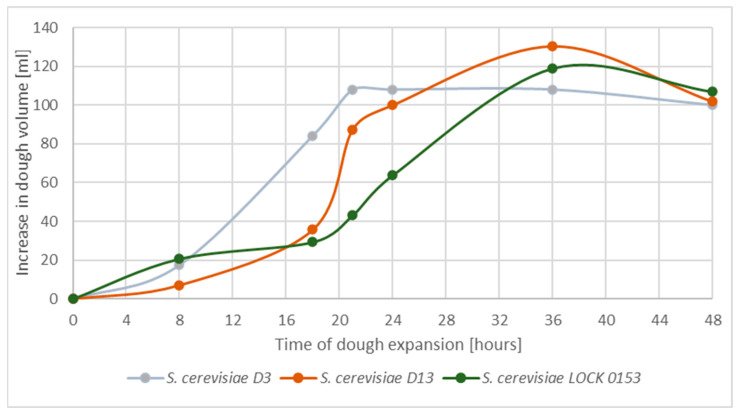
Production of carbon dioxide during yeast fermentation of wheat dough at 15 °C, presented as changes in the volume of wheat dough. The results were calculated per 100 mL of the initial volume of the sample.

To determine the productivity of carbon dioxide in wheat dough, the CO_2_ production rate and possible differences in the time of maximum production were investigated. Figure 4 shows the volume of carbon dioxide produced in ml per 100 mL of wheat dough.

Between hours 36 and 38 of the process conducted at 15 °C, *S. cerevisiae* D13 and *S. cerevisiae* LOCK 0153 reached their maximum carbon dioxide productivity. The sample with *Saccharomyces cerevisiae* D3 reached its maximum volume of dough in hour 24 of fermentation.

There is lack of research on the process of bread dough expansion at low temperatures. However, researchers have conducted similar but more detailed experiments on gas productivity during dough leavening. Chevallier and coworkers investigated different methods of measuring the expansion of bread dough during fermentation, in order to describe the variation in dough volumes at 25 °C, 30 °C, and 35 °C. They discovered a correlation between the time of fermentation, gas productivity, and the temperature of the process. They concluded that carbon dioxide production increased with the time at each temperature and reached its highest value at 35 °C [37]. Another interesting study was conducted by Ktenioudaki and coworkers, who investigated the aeration profile and baking quality of wheat dough. Using a standard temperature of dough leavening (35 °C) and with the incorporation of traditional bakery yeasts, they observed the highest gas productivity after around 100 min of fermentation [38].

#### 2.2.2. Carbon Dioxide Production During Mixed Culture Fermentation

Mixed culture fermentation improves the nutritional value of bread as well as its functional properties [39]. Due to the incorporation of both yeast and lactic acid bacteria, the bioavailability of components such as dietary fibre, amino acids, and proteins is increased [40]. Moreover, sourdough lowers the glycaemic index of bakery products and makes microelements more available [41]. Mixed cultures also improve the characteristics of the dough, which shows better gas retention during fermentation [40].

**Figure 5:**
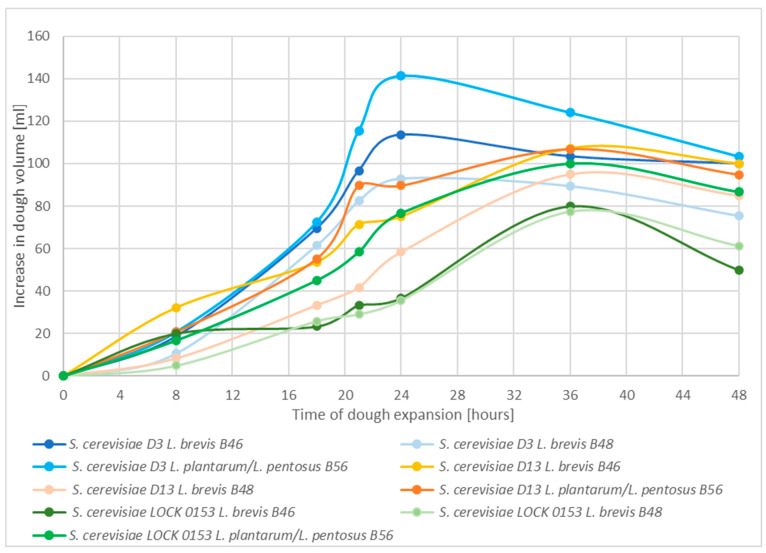
Production of carbon dioxide during mixed culture fermentation of wheat dough at 15 °C, presented as changes in volume of the wheat dough. Results calculated per 100 mL of the initial volume of the sample.

Figure 5 shows the increase in dough volume during fermentation by mixed cultures of yeast and lactic acid bacteria.

The highest possible productivity of CO_2_ was obtained for mixed culture *Saccharomyces cerevisiae* D3 and *L. plantarum*/*L. pentosus* B56 at around 140 mL of dough volume. It can be concluded that the use of lactic acid bacteria improved the volume of the dough. This may be due to the enzymatic activity of the bacteria. Sugars are released that are then used by the yeast, resulting in the increased production of carbon dioxide bubbles [42]. The samples containing *S. cerevisiae* D13 and lactic acid bacteria showed a lower production of carbon dioxide during fermentation at 15 °C. The same observation was made for yeast from the pure culture collection *S. cerevisiae* LOCK 0153. In these samples, dough expansion was limited when lactic acid bacteria were present during fermentation.

#### 2.2.3. Fermentation Activity of Yeasts and Lactic Acid Bacteria—Production of Ethanol, Acetic Acid, and Lactic Acid

The fermentation activity of microorganisms during the dough expansion process was assessed based on the production of lactic acid, acetic acid, and ethanol by mixed cultures. These metabolites are the main fermentation products of the yeasts and lactic acid bacteria and have a positive effect on the quality of the final product. Lactic acid produced by lactic acid bacteria contributes to the flavour of bread and lowers the pH, increasing shelf-life [43]. Acetic acid is a metabolite of both yeast and lactic acid bacteria. It also contributes to the taste of bread and lowers the pH of the dough, limiting the growth of unwanted microflora [39]. Although almost all ethanol evaporates during baking, it has a beneficial effect on the texture of the dough after fermentation [44].

Table 3 shows the concentrations of the primary metabolites of yeast and heterofermentative lactic acid bacteria produced during wheat dough expansion.

The data show the fermentation activity of three *Saccharomyces cerevisiae* strains (D3, D13, LOCK 0153) in combination with three lactic acid bacteria strains (*L. brevis* B46, *L. brevis* B48, and *L. plantarum*/*pentosus* B56). As can be seen, the combination of *S. cerevisiae* D3 and *L. brevis* B46 yielded the highest amounts of L-lactic acid (1.24 g/100 g), acetic acid (0.71 g/100 g), and ethanol (0.43 g/100 g), indicating strong metabolic activity. In contrast, the combination of *S. cerevisiae* D3 with *L. brevis* B48 produced the highest D-lactic acid (0.63 g/100 g) but the lowest ethanol concentration (0.18 g/100 g). It is worth noticing that the production of L-lactic acid was higher in almost all of the tested samples. D-lactic acid was produced in relatively smaller amounts. These results correspond to other research, in which the concentrations of these metabolites were found to be at similar levels [45,46,47]. Overall, *S. cerevisiae* D3 combined with *L. brevis* B46 appears to be the most effective at enhancing the production of primary metabolites that contribute to the flavour, texture, and preservation qualities of fermented bread.

#### 2.2.4. Formation of Volatile Organic Compounds in Wheat Doughs

The fermentation of wheat dough results in the production of several types of volatile organic compounds. Many of these compounds play a role in the development of dough aroma and flavour, which are directly related to the sensory quality of the final baked product. Table 4 indicates the aroma defined by the sense of smell.

The analysis of sensory impressions highlights the significant influence of different groups of aroma compounds on the odour profile of wheat dough. In samples where aromas were described as “fusel, pungent”, for instance *S. cerevisiae* D13 and *L. brevis* B46, this indicates the presence of significant amounts of higher alcohols. Wheat doughs with the addition of *L. plantarum*/*L. pentosus* B56 exhibited fruity notes (“fruity, apple”), which can be attributed to the presence of esters like ethyl acetate and compounds such as 2-phenylethanol, known for their floral aromas. Additionally, the alcoholic odour reported in samples is linked to the presence of ethanol. On the other hand, the sourdough-like aroma refers to more than one compound. It is the result of the interaction of those compounds, which in appropriate proportions create a characteristic sour, fermentative aroma reminiscent of traditional sourdough bread.

Yeasts and lactic acid bacteria produce primary metabolites during fermentation, such as ethanol and organic acids, but also other aroma compounds including carbonyl compounds and esters.

Volatile organic compounds were analysed in wheat doughs fermented at 15 °C. Nine variants of wheat doughs were studied, inoculated with different mixed bacterial and yeast cultures. The results are presented in Table 5.

The analysis reveals significant differences in the production of carbonyl compounds, higher alcohols, and esters across various dough variants with different combinations of *S. cerevisiae* and lactic acid bacteria strains. These VOCs play a crucial role in determining the aroma, flavour, and overall sensory quality of the dough and the final baked product.

Hexanal, a prominent carbonyl compound, varies noticeably across the different dough variants. The highest concentration was observed in the combination of *S. cerevisiae* D3 and *L. plantarum*/*L. pentosus* B56, which is known to contribute to a fresh and grassy aroma. In contrast, the combination of *S. cerevisiae* D13 and *L. brevis* B48 yields a significantly lower hexanal concentration, which may result in a milder aroma profile. Lactic acid bacteria had no consistent impact on the production of this compound.

Among the higher alcohols, 2-phenylethanol showed particularly high concentrations, especially for the combination of *S. cerevisiae* D13 with *L. brevis* B48. This compound is known for its pleasant rose-like aroma and is a key contributor to the overall fragrance of the dough. The high concentrations of 2-phenylethanol in this dough suggest a robust amino acid metabolism, specifically the Ehrlich pathway, in which aromatic amino acids are converted into higher alcohols [48]. Additionally, 3-methylbutanol and 2-methyl-1-propanol exhibited significant variability. The combination of *S. cerevisiae* D3 and *L. plantarum*/*L. pentosus* B56 led to the highest production of 3-methylbutanol, which is associated with fruity aromas, enhancing the flavour complexity of the dough. Other higher alcohols, 2-ethyl-1-hexanol, 2-methylbutanol, 1-hexanol, and 1-octanol, were also present in the wheat doughs after fermentation. However, the biosynthesis of 1-octanol was not detected in three variants of wheat dough fermented by *S. cerevisiae* D13.

Turning to esters, 14 compounds were identified. Ethyl acetate was detected in the highest concentrations in the dough containing *S. cerevisiae* D3 and *L. plantarum*/*L. pentosus* B56. The lowest concentration was found for the sample containing *S. cerevisiae* D13 and *L. brevis* B46. Ethyl acetate is known for its fruity, sweet aroma and is a major ester contributing to the sensory attributes of bread. Other esters, including ethyl hexanoate, were present in significant amounts in the doughs fermented by *S. cerevisiae* D3 with lactic acid bacteria. Ethyl formate was found in the highest concentrations in the samples fermented by *S. cerevisiae* LOCK0153 and lactic acid bacteria. Ethyl isobutyrate, 2-methylbutyl acetate, and ethyl 2-methylbutyrate were not quantified at all in doughs with the addition of *S. cerevisiae* D13 and *S. cerevisiae* LOCK0153. Other esters were detected in rather small concentrations, which also contributed fruity notes to the aroma profile. Based on these results, the mixed culture of *S. cerevisiae* D3 and *L. brevis* B46 and *S. cerevisiae* D3 and *L. plantarum*/*L. pentosus* B56 provided the most diverse aroma profile of the wheat dough. These two combinations of microorganisms also presented the most effective carbon dioxide production which related to the best results for dough leavening.

Various production techniques and procedures are employed in the baking industry to improve quality while simultaneously optimising costs. Low-temperature fermentation is gradually gaining popularity, due to its ability to slow down the fermentation process, allowing for the development of a more complex and thermally stable aroma profile. Additionally, it can reduce production costs by using lower temperatures during fermentation, leading to decreased electricity consumption [14]. Low-temperature processes can be applied to the fermentation of sourdough and dough leavening, where mixed cultures of yeasts and lactic acid bacteria play a crucial role in achieving a balanced aroma profile and enhancing the texture of the final product. Slow fermentation at lower temperatures promotes the biosynthesis of volatile organic compounds, such as esters, alcohols, and organic acids, which play a crucial role in determining the sensory attributes of bread [16]. Furthermore, the proper amounts of organic acids are important for prolonging the shelf-life of the product [49].

Although the interactions between different microbial strains have been described in the literature, more work is needed on their optimisation during low-temperature fermentation to enhance specific sensory qualities of bread. For instance, in this study, using *Saccharomyces cerevisiae* in combination with lactic acid bacteria such as *Levilactobacillus brevis* or *Lactiplantibacillus plantarum* at temperatures of around 10 °C to 15 °C significantly improved the aroma and flavour of bread by increasing the production of key VOCs. These findings are supported by research showing that lower fermentation temperatures can lead to higher concentrations of desirable flavour compounds, such as ethyl acetate and 2-phenylethanol, as well as other esters that contribute to pleasant taste and aroma [16]. The use of low-temperature fermentation is not only limited to improving flavour but also plays a crucial role in extending the shelf-life of bread. The proper selection of microbial strains used in low-temperature fermentation is also crucial for achieving the desired characteristics of the final product. The combination of yeast with specific strains of lactic acid bacteria can result in the production of key flavour compounds, while also enabling the simultaneous production of carbon dioxide, which is essential for dough leavening and the development of desirable texture properties. It is also essential for balancing the biosynthesis of carbonyl compounds, higher alcohols, and esters, which contribute to the high quality and customer acceptance of the final product [7].

## 3. Materials and Methods

### 3.1. Raw Material

Wheat flour type 650 was used as a raw material.

### 3.2. Chemical Reagents

The following chemicals, reagents, and analytical standards were used in this study:

- Wort broth (Merck, Darmstadt, Germany),
- MRS (DE MAN, ROGOSA and SHARPE) broth (Merck, Darmstadt, Germany),
- Agar,
- API^®^ ZYM (bioMérieux, Marcy-l’Étoile, France),
- ZYM A, ZYM B reagents (bioMérieux, Marcy-l’Étoile, France),
- D-/L-Lactic Acid (D-/L-Lactate) rapid test kit (Megazyme, Bray, Ireland),
- Acetic Acid Assay Kit (Megazyme, Bray, Ireland),
- The Ethanol test kit (Megazyme, Bray, Ireland),
- Nystatine,
- Gentamicin,
- Carrez I, Carrez II reagents,
- Sodium chloride,
- Sulphuric acid,
- Analytical standards for high-performance liquid chromatography: Glucose, fructose, maltose, maltotriose, arabinose,
- Analytical standards for gas chromatography: Hexanal, 2-Ethyl-1-Hexanol, 1-Octanol, 2-Methyl-1-Propanol, 3-Methylbutanol, 2-Methylbutanol, 1-Hexanol, 2-Phenylethanol, Ethyl propionate, Ethyl formate, Ethyl acetate, Ethyl valerate, Ethyl isobutyrate, Hexyl acetate, Ethyl butyrate, Ethyl nonanoate, Ethyl decanoate, 3-Methylbutyl acetate, 2-Methylbutyl acetate, Ethyl hexanoate, Ethyl 2-methylbutyrate, Ethyl octanoate.

### 3.3. Microbial Biomass

#### 3.3.1. Yeast Strains and Cultivation

Five environmental *Saccharomyces cerevisiae* isolates were investigated. Three of the isolates were environmental *Saccharomyces* isolates, and two were from the LOCK pure culture collection. Adaptation to the low temperature was carried out by multiple passaging of pure cultures at decreasing temperatures. The yeasts were selected based on their growth rate at 15 °C, indicated as optical density and CFU/mL (Supplementary Materials: Figures S1 and S2), as well as based on their metabolic activity for assimilating sugars (Supplementary Materials: Table S1) and producing volatile organic compounds in the model medium (Supplementary Materials: Table S2).

Three *Saccharomyces* yeast strains were used in the study. The strains were cultured in 10 mL of wort broth (Merck, Darmstadt, Germany) with gentamycin in test tubes at 15 °C for 5 days. All the environmental isolates were deposited in the pure culture collection at the Department of Environmental Biotechnology at Lodz University of Technology in Poland (Table 6).

#### 3.3.2. Lactic Acid Bacteria Strains and Cultivation

Three bacterial strains were used in this study. The strains were selected from 11 isolates. All of the isolates were environmental strains previously isolated from wheat flour and adapted to low temperature. They were cultured in 10 mL of MRS (DE MAN, ROGOSA, and SHARPE) broth (Merck, Darmstadt, Germany) with nystatin in test tubes at 15 °C for 5 days. All the isolates were deposited in the pure culture collection at the Department of Environmental Biotechnology at Lodz University of Technology in Poland (Table 7).

### 3.4. Enzymatic Activity Assessed by API ZYM Tests

The enzymatic activity of the tested yeast and lactic acid bacteria isolates was evaluated by API ZYM (bioMérieux, Marcy-l’Étoile, France), according to the manufacturer’s instructions. The aim of the analysis was to evaluate the potential utilization of carbohydrates present in wheat flour. API ZYM is a semi-quantitative method for the investigation of enzymatic activity. The technique is applicable to all types of samples (microorganisms, cell suspensions, tissues, biological fluids, etc.). It enables the systematic and rapid study of 19 enzymatic reactions using very small sample quantities. The examined yeast and lactic acid bacteria were subjected to analysis of enzymatic activity at 15 °C.

### 3.5. Sponge Fermentation

To determine the ability of yeast to grow in a wheat sponge at 15 °C, selected strains were cultured as described in Section 2.1. Wheat flour–water mixture was inoculated with three different amounts of inoculum. First, yeast biomass cultured in wort broth at 15 °C was centrifuged at 4200 rpm for 10 min. Then, the supernatant was removed, the yeast biomass was suspended in distilled water, and the concentration was adjusted to 10^7^ CFU/mL).

Sponge starters were prepared by mixing 40 g of wheat flour with 40 mL of water, then inoculated with prepared yeast suspensions at concentrations of 1%, 5%, and 10% inoculum. The exact number of CFU/mL in the sourdough starters was determined by the pour plate method at 15 °C for 5 days in wort broth with gentamycin.

A mixture containing 40 g of wheat flour and 40 mL of water was used as a control sample. Both the inoculated sponge starters and the control sample were incubated at 15 °C for 5 days. After this time, the final CFU/mL in each sample and the control were determined by the pour plate method at 15 °C for 5 days in wort broth with gentamycin.

### 3.6. Quantitative Analysis of Sugar Content Using HPLC

Prior to analysis, the samples were clarified using Carrez reagents, centrifuged, and filtered through a syringe filter with a 0.45 μm PES (polyethersulfone) membrane. The content of maltotriose, maltose, glucose, fructose, and arabinose in the supernatants was determined using a high-performance liquid chromatograph (HPLC) 1260 Infinity (Agilent Technologies, Santa Clara, CA, USA) with refractometric detection (RID). The samples were injected in a volume of 20 μL and the sugars were separated using a Hi-Plex H column (Agilent Technologies, Santa Clara, CA, USA) at a temperature of 60 °C. The mobile phase used was 0.005 M H_2_SO_4_ with a flow rate of 0.7 mL/min. The temperature of the detector was set at 55 °C. The chromatograms obtained were analysed using OpenLab CDS Chemstation software Rev. C.01.06 (Agilent Technologies, Santa Clara, CA, USA). The concentration of each sugar was calculated from the standard curves for individual compounds.

### 3.7. Ethanol, Acetic Acid, and Lactic Acid Content Analysis

To measure the ethanol, acetic acid, and lactic acid concentrations, assay kits from Megazyme (Bray, Ireland) were used. The tests were conducted according to the manufacturer’s instructions. All tests were performed on 96-well plates using a spectrophotometer (Thermo Scientific Multiskan GO, Thermo Fisher Scientific, Waltham, MA, USA).

### 3.8. Productivity of Carbon Dioxide

The fermentation activity of the selected yeast strains was measured and expressed as the dough expansion. The potential intensification of fermentation was tested by the application of lactic acid bacteria with the selected yeast strains. First, yeast and lactic acid bacteria were centrifuged at 4200 rpm for 10 min. Then, biomass suspensions were prepared by adding distilled water and adjusting the concentration of yeasts to 10^7^ CFU/mL and lactic acid bacteria to 10^8^ CFU/mL. Next, 40 g of wheat flour was mixed with 12.5 mL of yeast suspension and 12.5 mL of tap water. In the case of mixed cultures, 40 g of wheat flour was mixed with 12.5 mL of yeast suspension and 12.5 mL of bacterial suspension. The dough was formed, placed in a volumetric cylinder, and covered with aluminum foil to prevent the dough surface from drying. The initial volume of each dough was noted. The doughs were incubated at 15 °C for 48 h and the volume change during incubation was measured.

### 3.9. Sensory Analysis of Aroma in Wheat Doughs

In this study, sensory analysis refers to the description of aroma in wheat doughs fermented at 15 °C. For this purpose, doughs obtained according to point 4.7 were assessed. The evaluation panel was formed of 10 people, including employees, PhD candidates, and diploma students from the Department of Environmental Biotechnology, Faculty of Biotechnology and Food Sciences, Lodz University of Technology. Each person identified and assessed the fragrance of each sample using the sense of smell. After examination of all wheat doughs, all the observations were summarised in a table.

### 3.10. Quantitative Analysis of Volatile Organic Compounds Biosynthesis Using the HS-SPME-GC-MS Technique

Volatile organic compounds were identified by gas chromatography coupled with mass spectrometry (GC-MS) using static headspace extraction (HS). Analyses were carried out using an Agilent Technologies model 7890A (Santa Clara, CA, USA) gas chromatograph coupled to an Agilent Technologies MSD 5975C (Santa Clara, CA, USA) mass spectrometer and an Agilent Technologies 7697A (Santa Clara, CA, USA) headspace sampler. To a 20 mL glass vial were added 1 g of the sample and 9 g of water, which were mixed thoroughly to obtain a homogeneous suspension. Headspace conditions were as follows: oven temperature 50 °C; loop temperature 60 °C; transfer line temperature 70 °C; vial equilibration time 20 min; injection duration 0.7 min. During extraction, the vial was shaken at 136 shakes/min with acceleration of 530 cm/s^2^.

Separation of compounds was carried out on an Rxi-5MS 60 m × 250 μm × 0.25 μm (Restek, Bellefonte, PA, USA) capillary column with dimensions of 60 m (length) × 250 μm (internal diameter) × 0.25 μm (thickness of the stationary phase). The temperature program for the oven began at 30 °C, was held for 5 min, then were increased at a rate of 2 °C/min to 60 °C, held for 1 min, followed by an increase at 20 °C/min to 240 °C, where it was held for 8 min. The flow rate of the carrier gas (helium) through the column was 1 mL/min. The temperature of the GC injector was 250 °C. The temperature of the transfer line to MS was 250 °C. The temperature of the ion source was 230 °C. The temperature of the quadrupole was 150 °C. During analysis, the mass spectrometer was operated in the full scan mode (SCAN) for the identification of volatiles and then in the ion monitoring mode (SIM) for the quantification of volatiles. The obtained data were analysed using Agilent MassHunter software, version 12.1 (Agilent Technologies, Santa Clara, CA, USA).

### 3.11. Statistical Analysis

HS-GC-MS analyses were performed in triplicate. Statistical analyses were performed using XLSTAT software Addinsoft, version 2022.2.1, USA. The obtained results were evaluated using analysis of variance (ANOVA, at the 0.05 significance level) to indicate differences. If statistical differences were detected (*p* < 0.05), means were compared by Tukey’s test (at the 0.05 significance level).

PCA was used to determine the best differentiation of volatile compounds in the wheat sponge fermented by three yeast strains.

## 4. Conclusions

Based on the result of this study, the following conclusions can be drawn:

- All tested yeast and lactic acid bacteria strains demonstrated enzymatic activity at 15 °C, which enabled the efficient biosynthesis of volatile organic compounds.
- All tested yeast strains presented growth in a mixture of flour and water at 15 °C.
- The sponge samples fermented by *S. cerevisiae* D3 were characterised by the highest concentration of volatile organic compounds, especially esters.
- The highest carbon dioxide productivity at 15 °C was recorded for the samples fermented by mixed cultures of *S. cerevisiae* D3 and *L. plantarum*/*L. pentosus* B56, as well as *S. cerevisiae* D3 and *L. brevis* B46.
- Wheat dough fermented by the mixed culture of *S. cerevisiae* D3 and *L. brevis* B46 was characterised by the highest ester content.
- Low-temperature fermentation is a promising technique to produce wheat dough with enhanced flavour and desirable texture obtained by dough leavening.

Ongoing research into the interactions between various microbial strains and the optimization of fermentation conditions is essential for developing high-quality products.

## Figures and Tables

**Table 1:** Enzymatic activity of tested yeasts at 15 °C measured by API ZYM tests.

	*S. cerevisiae* D3	*S. cerevisiae* D13	*S. cerevisiae* LOCK0153
Alkaline phosphatase	2	1	1
Esterase (C4)	2	2	3
Esterase Lipase (C8)	1	1	2
Leucine arylamidase	5	5	5
Valine arylamidase	4	4	3
Cystine arylamidase	1	1	3
Acid phosphatase	5	5	5
Naphthol-AS-BI-phosphohydrolase	5	5	5
α-glucosidase	0	0	3

Numbers in columns indicate the intensity of colouration on a 5-point scale (from 0 to 5): 0—no reaction, 1—weak reaction, 2—moderate reaction, 3—strong reaction, 4—very strong reaction, 5—extremely strong reaction.

**Table 2:** Enzymatic activity of tested lactic acid bacteria at 15 °C measured by API ZYM tests.

	*L. brevis* B46	*L. brevis* B48	*L. plantarum*/ *L. pentosus* B56
Esterase (C4)	3	2	0
Esterase Lipase (C8)	2	1	0
Leucine arylamidase	5	5	5
Valine arylamidase	4	4	5
Cystine arylamidase	3	3	3
Acid phosphatase	4	4	2
Naphthol-AS-BI-phosphohydrolase	3	3	2
α-galactosidase	4	3	2
ß-galactosidase	5	5	5
ß-glucuronidase	0	2	0
α-glucosidase	3	3	4
ß-glucosidase	5	5	5
N-acetyl-ß-glucosaminidase	0	0	5

Numbers in columns indicate the intensity of colouration on a 5-point scale (from 0 to 5): 0—no reaction, 1—weak reaction, 2—moderate reaction, 3—strong reaction, 4—very strong reaction, 5—extremely strong reaction.

**Table 3:** Ethanol, acetic acid, and lactic acid content in wheat dough after fermentation at 15 °C.

	*Saccharomyces cerevisiae* D3			*Saccharomyces cerevisiae* D13			*Saccharomyces cerevisiae* LOCK 0153		
	*L. brevis* B46	*L. brevis* B48	*L. plantarum*/ *L. pentosus* B56	*L. brevis* B46	*L. brevis* B48	*L. plantarum*/ *L. pentosus* B56	*L. brevis* B46	*L. brevis* B48	*L. plantarum*/ *L. pentosus* B56
	Concentration [g/100 g]								
D-lactic acid	0.51 c ± 0.06	0.63 d ± 0.06	0.59 e ± 0.1	0.39 a ± 0.04	0.62 b ± 0.01	0.32 i ± 0.05	0.51 f ± 0.04	0.53 g ± 0.01	0.44 h ± 0.06
L-lactic acid	1.24 b ± 0.23	0.82 g ± 0.1	0.63 c ± 0.11	0.77 a ± 0.03	0.63 f ± 0.01	0.65 i ± 0.03	0.57 d ± 0.05	0.51 e ± 0.09	0.83 h ± 0.02
Acetic acid	0.71 b ± 0.08	0.52 c ± 0.0	0.56 d ± 0.0	0.4 a ± 0.02	0.46 g ± 0.13	0.47 h ± 0.06	0.63 e ± 0.11	0.38 i ± 0.02	0.55 f ± 0.06
Ethanol	0.43 d ± 0.03	0.18 e ± 0.0	0.28 f ± 0.02	0.07 a ± 0.02	0.31 b ± 0.0	0.14 c ± 0.01	0.17 g ± 0.03	0.13 h ± 0.0	0.13 i ± 0.01

a–i—mean values in columns denoted by different letters differ statistically significantly (one-way ANOVA, *p* < 0.05).

**Table 4:** Descriptive sensory analysis of wheat doughs fermented with mixed cultures at 15 °C.

	*L. brevis* B46	*L. brevis* B48	*L. plantarum*/*L. pentosus* B56
*S. cerevisiae* D3	alcoholic, sourdough-like, slightly floral	sourdough-like	fruity, apple, fusel
*S. cerevisiae* D13	fusel, pungent	fruity, fusel, sourdough-like	fruity, pungent
*S. cerevisiae* LOCK 0153	sourdough-like, fusel	fruity, fusel	alcoholic, fruity

**Table 5:** Formation of volatile organic compounds in wheat doughs fermented by the mixed cultures.

	*Saccharomyces cerevisiae* D3			*Saccharomyces cerevisiae* D13			*Saccharomyces cerevisiae* LOCK0153		
	*L. brevis* B46	*L. brevis* B48	*L. plantarum*/ *L. pentosus* B56	*L. brevis* B46	*L. brevis* B48	*L. plantarum*/ *L. pentosus* B56	*L. brevis* B46	*L. brevis* B48	*L. plantarum*/ *L. pentosus* B56
	Concentration [µg/kg]								
	Carbonyl compounds								
Hexanal	249.97 d ± 0.68	342.53 f ± 1.29	371.96 g ± 3.71	128.37 b ± 0.39	100.17 a ± 0.31	243.87 d ± 0.29	283.69 e ± 0.88	209.07 c ± 1.14	137.68 b ± 0.34
	Higher alcohols								
2-Ethyl-1-Hexanol	9.56 fg ± 0.47	7.79 de ± 0.26	10.68 g ± 0.31	6.57 bc ± 0.27	5.83 b ± 0.27	3.94 a ± 0.16	6.94 bcd ± 0.26	7.74 cd ± 0.35	8.88 ef ± 0.13
1-Octanol	44.10 b ± 1.70	55.21 c ± 0.60	70.10 d ± 1.91	nd	nd	nd	71.50 d ± 0.07	56.66 c ± 0.34	34.31 a ± 0.38
2-Methyl-1-Propanol	1297.56 e ± 69.89	1516.08 f ± 10.73	3563.23 g ± 11.68	315.15 bc ± 7.50	385.7 cd ± 0.45	493.92 d ± 5.04	219.17 ab ± 0.88	177.43 ab ± 5.40	167.45 a ± 1.33
3-Methylbutanol	10,997.67 e ± 28.99	11,037.86 e ± 42.97	128,38.19 f ± 29.40	823.33 a ± 8.11	998.15 ab ± 26.75	1595.52 b ± 15.83	3907.65 d ± 16.76	3607.93 d ± 24.13	2819.68 c ± 26.69
2-Methylbutanol	864.02 c ± 8.99	1094.22 d ± 12.63	2028.29 e ± 10.64	125.52 a ± 0.81	196.52 ab ± 8.87	196.10 ab ± 5.39	261.84 b ± 1.83	226.64 b ± 6.61	212.67 b ± 4.65
1-Hexanol	2066.96 cd ± 1.44	2138.25 de ± 7.16	2576.68 f ± 6.13	30.57 a ± 0.23	nd	199.60 b ± 1.01	2215.68 de ± 2.48	1985.21 c ± 22.92	2275.54 e ± 5.35
2-Phenylethanol	46,747.55 e ± 15.85	40,170.95 d ± 40.69	48,967.50 e ± 12.15	21,994.97 b ± 85.17	69,597.79 f ± 230.77	15,729.12 a ± 95.19	25,172.52 b ± 231.99	22,613.3 b ± 461.57	30,509.96 c ± 294.35
	Esters								
Ethyl propionate	26.91 e ± 0.35	39.10 f ± 0.21	22.32 d ± 0.66	nd	nd	1.56 a ± 0.04	9.09 b ± 0.19	12.25 c ± 0.04	10.23 b ± 0.10
Ethyl formate	99.80 g ± 1.02	35.75 c ± 0.46	48.09 d ± 1.31	27.14 b ± 0.69	15.87 a ± 0.46	20.36 a ± 0.52	55.89 e ± 1.10	67.45 f ± 0.60	70.32 f ± 0.65
Ethyl acetate	46,046.71 e ± 45.58	29,091.78 d ± 28.89	56,907.40 f ± 10.29	1345.06 a ± 30.08	2446.26 a ± 27.88	7207.35 b ± 0.37	8210.98 b ± 78.15	8858.57 b ± 52.04	14,972.2 c ± 26.17
Ethyl valerate	41.46 e ± 0.08	36.65 d ± 0.02	50.17 f ± 1.11	nd	nd	0.66 a ± 0.08	6.89 b ± 0.59	7.24 bc ± 0.24	9.33 c ± 0.33
Ethyl isobutyrate	nd	0.89 a ± 0.03	1.73 b ± 0.04	nd	nd	nd	nd	nd	nd
Hexyl acetate	7.88 e ± 0.04	2.22 b ± 0.02	1.64 a ± 0.05	2.59 b ± 0.05	nd	nd	5.01 c ± 0.05	2.30 b ± 0.08	7.09 d ± 0.19
Ethyl butyrate	6.40 b ± 0.25	6.93 c ± 0.17	6.55 b ± 0.19	nd	nd	nd	nd	nd	1.30 a ± 0.07
Ethyl nonanoate	2.68 c ± 0.03	2.31 b ± 0.01	2.49 b ± 0.08	nd	nd	nd	0.36 a ± 0.01	0.40 a ± 0.03	0.39 a ± 0.01
Ethyl decanoate	3.31 b ± 0.08	3.30 b ± 0.01	3.73 c ± 0.12	nd	nd	nd	0.20 a ± 0.01	0.21 a ± 0.03	0.14 a ± 0.01
3-Methylbutyl acetate	6.73 c ± 0.20	1.33 a ± 0.10	4.38 b ± 0.11	nd	1.02 a ± 0.04	nd	1.27 a ± 0.08	nd	1.37 a ± 0.07
2-Methylbutyl acetate	1.33 ± 0.04	nd	nd	nd	nd	nd	nd	nd	nd
Ethyl hexanoate	603.85 d ± 1.87	582.53 d ± 1.40	547.38 c ± 2.32	4.17 a ± 0.06	4.26 a ± 0.04	6.44 a ± 0.09	80.74 b ± 0.61	98.84 b ± 1.29	101.98 b ± 4.19
Ethyl 2-methylbutyrate	0.56 b ± 0	0.40 a ± 0.04	0.61 b ± 0.01	nd	nd	nd	nd	nd	nd
Ethyl octanoate	57.54 c ± 0.46	57.65 c ± 0.03	60.73 c1.50	0.68 a ± 0.04	0.89 a ± 0.03	1.04 a ± 0.01	4.74 b ± 0.1	5.32 b ± 0.14	4.79 b ± 0.18

nd—not detected; a–f—mean values in columns denoted by different letters differ statistically significantly (one-way ANOVA, *p* < 0.05).

**Table 6:** Yeast isolates. The selected strains are highlighted in yellow.

Isolate No.	Species Identification
D2	*Saccharomyces cerevisiae*
D3	*Saccharomyces cerevisiae*
D13	*Saccharomyces cerevisiae*
	LOCK pure culture collection
LOCK 0157	*Saccharomyces cerevisiae*
LOCK 0153	*Saccharomyces cerevisiae*

**Table 7:** Lactic acid bacteria isolates. The selected strains are highlighted in blue.

Isolate No.	Species Identification
B1	*Levilactobacillus brevis*
B5	*Levilactobacillus brevis*
B6	*Levilactobacillus brevis*
B16	*Pediococcus acidilactici*
B17	*Lactiplantibacillus plantarum*
B20	*Levilactobacillus brevis*
B46	*Levilactobacillus brevis*
B48	*Levilactobacillus brevis*
B50	*Leuconostoc citreum*
B56	*Lactiplantibacillus plantarum*/*Lactiplantibacillus pentosus*
B64	*Lactiplantibacillus plantarum*/*Lactiplantibacillus pentosus*

## Data Availability

Data is contained within the article or Supplementary Materials.

## Supplementary Materials

The following supporting information can be downloaded at https://www.mdpi.com/article/10.3390/molecules30010112/s1: Figure S1: Growth dynamics expressed as optical density of yeast suspensions at 15 °C; Figure S2: Growth dynamics expressed as CFU/mL of yeast suspensions at 15 °C; Figure S3: Growth dynamics expressed as optical density of lactic acid bacteria suspensions at 15 °C; Table S1: Assimilation of sugars conducted by API 20 C AUX at 15 °C. Abbreviations: GLU—D-glucose; 2KG—calcium 2-Keto-Gluconate; GAL—D-galactose; CEL—D-cellobiose; MAL—D-maltose; “SAC”—D-saccharose; RAF—D-raffinose; Table S2: Quantitative analysis of volatile organic compounds biosynthesis in the model medium at 15 °C; Table S3: Quantitative analysis of esters biosynthesis in the wheat sponge fermented at 15 °C. Abbreviations: “Control” indicates control sample before fermentation; “SF” indicates spontaneous fermentation; Table S4: Quantitative analysis of higher alcohols biosynthesis in the wheat sponge fermented at 15 °C. Abbreviations: “Control” indicates control sample before fermentation; “SF” indicates spontaneous fermentation; Table S5: Quantitative analysis of carbonyl compounds biosynthesis in the wheat sponge fermented at 15 °C. Abbreviations: “Control” indicates control sample before fermentation; “SF” indicates spontaneous fermentation.
