# Supplementary material for: Mixed Culture of Yeast and Lactic Acid Bacteria for Low-Temperature Fermentation of Wheat Dough
Source: Molecules. 2024 Dec 30;30(1):112. doi: 10.3390/molecules30010112 (PMC11722544; doi:10.3390/molecules30010112)
Supplement: Supplementary file 1 [file molecules-30-00112-s001.zip › molecules-3337753-supplementary.pdf]

SUPPLEMENTARY MATERIALS

All the yeast and bacteria environmental isolates were characterized and deposited in the pure culture collection at the Department of Environmental Biotechnology of the Lodz University of Technology in Poland. Assimilation profiles and growth dynamic for *S. cerevisiae* D2 and *S. cerevisiae* D13 were previously published (DOI accession number: 10.15376/biores.18.1.599-612).

YEAST

Table S1. Assimilation of sugars conducted by API 20 C AUX at 15℃. Abbreviations: GLU - D-glucose; 2KG - calcium 2-Keto-Gluconate; GAL - D-galactose; CEL - D-cellobiose; MAL - D-maltose; SAC - D-saccharose; RAF - D-raffinose.

Isolate no.	Assimilated sugars						
	GLU	2KG	GAL	CEL	MAL	SAC	RAF
S. cerevisiae D2	+	-	+	-	-	+	+
S. cerevisiae D3	+	-	+	-	+	+	-
S. cerevisiae D13	+	-	-	-	-	+	-
S. cerevisiae LOCK 0157	+	+	-	-	-	-	-
S. cerevisiae LOCK 0153	+	+	-	+	-	-	-

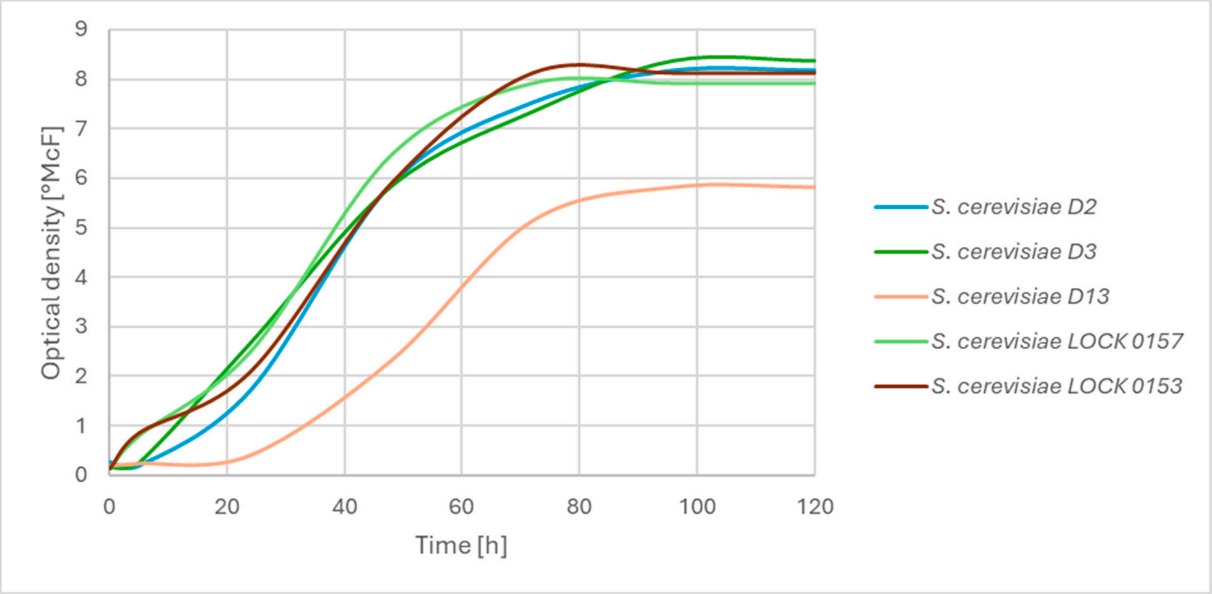

Figure S1. Growth dynamics expressed as optical density of yeast suspensions at 15°C.

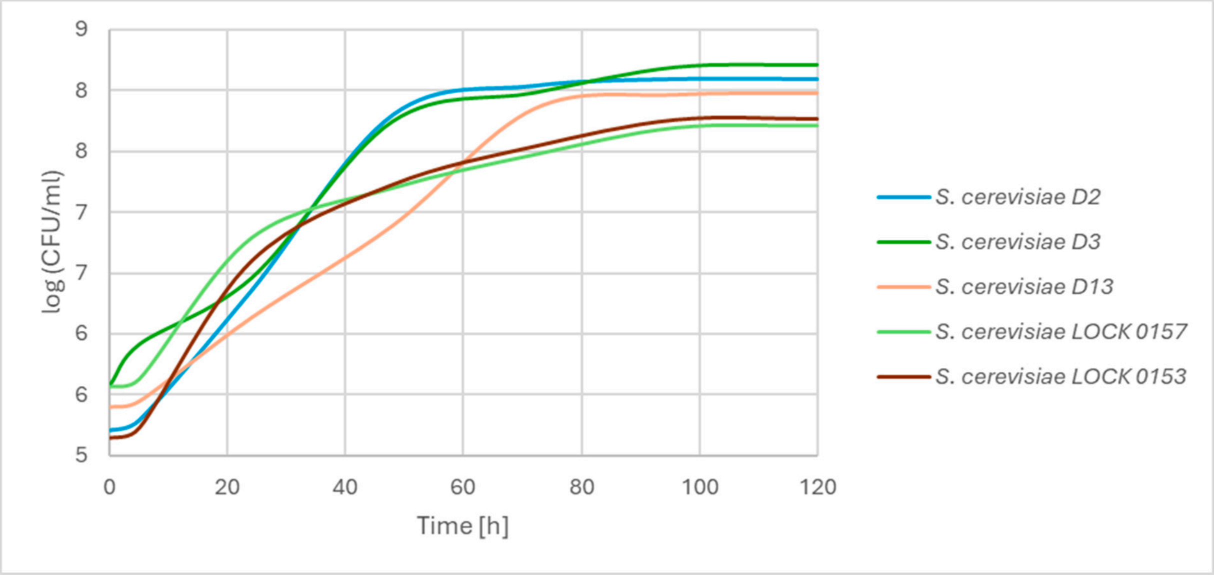

Figure S2. Growth dynamics expressed as CFU/ml of yeast suspensions at 15°C.

Table S2. Quantitative analysis of volatile organic compounds biosynthesis in the model medium at 15°C.

	S. cerevisiae D2	*S. cerevisiae* D3	*S. cerevisiae* D13	*S. cerevisiae* LOCK 0157	*S. cerevisiae* LOCK 0153
	Concentration [µg/l]				
	Carbonyl compounds				
Acetaldehyde	988.7±44.7	2388.1±118.6	749.6±42.2	972.8±30.7	2265.5±52.7
Acetone	185.6±4.0	80.1±2.0	81.2±8.4	54.0±5.0	90.8±2.4
Isobutyraldehyde	37.8±1.2	25.1±0.6	9.2±0.3	12.0±0.9	29.0±1.2
2,3-butanedione	82.4±9.9	79.4±2.8	55.3±5.1	46.1±4.8	37.6±2.6
Isovaleraldehyde	52±1.7	40.8±2.2	5.8±1.1	10.1±0.7	18.0±0.5
2-methylbutanal	21.5±0.8	10.0±0.3	3.0±0.1	5.3±0.2	7.8±0.2
	Higher alcohols				
3-methylbutanol	6903.2±172.2	12772.4±361.4	1063.8±33.7	8307.2±206.8	7543.8±176.9
2-methylbutanol	2299.4±49.6	3827.3±95.4	503.6±12.7	3153.3±136.2	2748.3±59.2
Isobutanol	1989.3±110.2	3259.0±91.8	563.6±17.2	4435.0±405.0	2811.0±59.0
Phenylethanol	4468.0±96.3	7382.9±733.9	397.9±29.3	3149.5±188.0	4542.8±114.8
	Esters				
Ethyl acetate	125.3±6.2	1298.1±33.4	16976.2±496.2	391.9±16.3	656.5±13.9
Ethyl propionate	1.2±0.2	5.5±0.1	5.6±0.6	2.1±0.0	6.4±0.2
Isobutyl acetate	0.9±0.2	2.1±0.2	0.4±0.0	1.4±0.1	2.2±0.0
Ethyl butyrate	2.6±0.3	6.6±0.4	1.9±0.1	1.8±0.1	1.2±0.0
3-methylbutyl acetate	2.9±0.1	24.8±1.5	1.6±0.1	10.4±0.4	25.1±0.5
2-methylbutyl acetate	0.3±0.1	2.7±0.1	0.4±0.0	1.3±0.0	3.3±0.1
Ethyl hexanoate	5.6±0.2	101.7±2.8	0.5±0.0	10.2±0.2	7.4±0.2
Ethyl heptanoate	0.7±0.1	0.7±0.0	0.1±0.0	0.2±0.0	0.2±0.0
Ethyl octanoate	6.8±0.1	858.8±53.7	0.6±0.0	69.2±1.7	85.3±2.4
2-phenylethyl acetate	25.1±4.2	40.0±2.0	2.5±0.1	12±0.5	23.4±0.6
Ethyl nonanoate	0.6±0.0	1.0±0.0	0.2±0.0	0.1±0.0	0.3±0.0
Ethyl decanoate	1.0±0.1	45.4±1.4	0.2±0.0	7.9±0.2	11±0.2
Ethyl dodecanoate	0.9±0.2	1.3±0.0	0.1±0.0	0.8±0.1	0.7±0.0

Table S3. Quantitative analysis of esters biosynthesis in the wheat sponge fermented at 15°C. Abbreviations: "Control" indicates control sample before fermentation; "SF" indicates spontaneous fermentation.

Sample	Inoculum		Concentration [µg/kg]																	
			Ethyl formate	Ethyl acetate	Ethyl propionate	Propyl acetate	Ethyl isobutyrate	Isobutyl acetate	Ethyl butyrate	Ethyl lactate	Butyl acetate	3-Methylbutyl acetate	2-Methylbutyl acetate	Ethyl hexanoate	Hexyl acetate	Isoamyl butyrate	Ethyl heptanoate	Ethyl octanoate	Ethyl nonanoate	Ethyl decanoate
Control	-	Mean	nd	2.43 j	nd	nd	nd	nd	nd	nd	nd	nd	nd	nd	nd	nd	nd	0.01 i	nd	nd
		SD	-	1.59	-	-	-	-	-	-	-	-	-	-	-	-	-	0.00	-	-
SF	-	Mean	nd	1414.66 i	0.55 g	0.30 f	nd	nd	304.34 a	24.32 a	14.22 a	0.21 e	nd	0.87 g	nd	0.82 a	nd	0.04 i	nd	nd
		SD	-	29.79	0.01	0.02	-	-	12.61	1.47	0.86	0.02	-	0.01	-	0.00	-	0.00	-	-
S. cerevisiae D3	1%	Mean	3.35 g	25941.28 a	4.97 b	10.18 a	0.27 c	3.26 a	7.42 fg	0.42 cd	0.33 cd	6.57 a	1.48 a	3.22 c	nd	nd	0.07 d	1.56 b	0.02 c	0.19 b
		SD	0.09	286.60	0.09	0.41	0.01	0.091	0.601	0.020	0.042	0.47	0.03	0.05	-	-	0.00	0.01	0.00	0.00
	5%	Mean	5.28 f	6758.96 e	8.56 a	2.13 d	0.57 a	0.59 e	13.93 ef	2.07 b	1.33 b	1.85 d	0.42 f	5.69 a	nd	0.09 e	0.12 a	2.21 a	0.04 a	0.22 a
		SD	0.35	224.22	0.21	0.03	0.02	0.04	0.14	0.08	0.01	0.04	0.02	0.06	-	0.00	0.00	0.03	0.00	0.01
	10%	Mean	3.39 g	10561.27 c	4.21 c	5.37 b	0.34 b	2.01 b	4.12 fg	1.02 bcd	0.74 bcd	3.60 b	1.08 b	3.43 b	0.08 a	nd	0.10 b	1.46 c	0.03 b	0.14 c
		SD	0.21	91.59	0.02	0.11	0.01	0.08	0.06	0.05	0.02	0.11	0.04	0.06	0.00	-	0.00	0.02	0.00	0.00
S. cerevisiae D13	1%	Mean	17.13 b	9961.23 d	1.71 e	2.04 d	0.16 d	0.79 d	40.53 bc	1.07 bcd	0.80 bcd	2.89 c	0.53 e	1.44 f	0.08 a	0.20 c	0.04 f	0.65 e	nd	0.05 e
		SD	0.59	75.85	0.03	0.08	0.00	0.04	0.39	0.07	0.063	0.06	0.00	0.01	0.01	0.01	0.00	0.00	-	0.00
	5%	Mean	14.25 c	3485.81 h	0.81 g	0.36 f	nd	0.13 f	24.900de	1.36 bcd	1.01 bc	0.35 e	0.05 h	0.46 h	nd	0.13 d	nd	0.22 h	nd	0.02 f
		SD	0.18	75.23	0.04	0.02	-	0.01	0.18	0.01	0.08	0.03	0.01	0.01	-	0.01	-	0.00	-	0.00
	10%	Mean	21.46 a	4629.39 g	1.91 e	3.72 c	nd	0.46 e	47.15 b	1.39 bcd	1.09 bc	2.75 c	0.29 g	1.38 f	nd	0.24 b	0.04 f	0.70 d	nd	0.07 de
		SD	0.28	17.65	0.05	0.02	-	0.00	0.18	0.02	0.02	0.02	0.00	0.01	-	0.00	0.00	0.01	-	0.00
S. cerevisiae LOCK 0153	1%	Mean	10.25 d	7054.32 e	2.19 d	2.30 d	0.31 b	0.91 d	29.22 cd	2.24 b	1.53 b	1.68 d	0.41 f	1.91 e	nd	0.08 f	0.05 e	0.58 f	nd	0.06 de
		SD	0.43	27.96	0.04	0.07	0.01	0.01	0.11	0.04	0.04	0.02	0.02	0.02	-	0.00	0.00	0.01	-	0.00
	5%	Mean	7.55 e	6020.20 f	1.13 f	1.42 e	0.15 d	1.28 c	4.28 fg	1.82 bc	1.19 bc	2.16 d	0.69 d	2.52 d	0.07 a	nd	0.08 c	0.71 d	nd	0.07 d
		SD	0.18	48.31	0.05	0.01	0.02	0.05	0.18	0.09	0.09	0.07	0.02	0.03	0.01	-	0.00	0.02	-	0.00
	10%	Mean	5.76 f	11072.76 b	1.79 e	3.94 c	nd	2.13 b	1.04 fg	nd	nd	3.02 c	0.77 c	1.34 f	nd	nd	0.03 f	0.51 g	nd	0.07 d
		SD	0.15	81.61	0.03	0.02	-	0.02	0.01	-	-	0.07	0.04	0.03	-	-	0.00	0.01	-	0.00

nd – not detected; a-g – mean values in columns denoted by different letters differ statistically significantly (two-way ANOVA, $p < 0.05$)

Table S4. Quantitative analysis of higher alcohols biosynthesis in the wheat sponge fermented at 15℃. Abbreviations: "Control" indicates control sample before fermentation; "SF" indicates spontaneous fermentation.

Sample	Inoculum		Concentration [µg/kg]						
			1-Propanol	2-Methyl-1-propanol	1-Butanol	3-Methylbutanol	2-Methylbutanol	1-Hexanol	2-Phenylethanol
Control	-	Mean	nd	nd	nd	nd	nd	9.96 h	nd
		SD	-	-	-	-	-	0.38	-
SF	-	Mean	24.56 h	21.95 g	6657.73 a	140.96 h	14.2 i	17.59 g	nd
		SD	0.70	0.20	95.46	4.70	1.10	0.07	-
S. cerevisiae D3	1%	Mean	2762.06 b	1562.92 c	25.39 b	6661.38 b	846.32 d	27.62 ef	2096.74 ab
		SD	37.42	25.06	10.52	32.30	4.72	0.86	777.17
	5%	Mean	2942.81 a	2759.61 a	23.71 b	10862.13 a	2498.98 a	65.68 c	2979.23 a
		SD	85.72	80.10	1.72	259.36	42.31	1.70	90.27
	10%	Mean	2085.76 c	1844.25 b	16.10 b	5832.81 c	1310.86 b	77.04 b	2411.46 a
		SD	8.26	7.41	0.26	53.30	9.88	1.79	159.71
S. cerevisiae D13	1%	Mean	354.67 g	519.75 e	30.55 b	3306.06 e	449.31 f	45.58 d	1163.80 bc
		SD	8.15	19.45	1.01	68.28	3.62	0.90	400.31
	5%	Mean	313.99 g	327.59 f	70.56 b	1307.52 g	201.63 h	15.18 gh	520.84 cd
		SD	2.06	7.97	0.61	9.08	3.91	0.37	24.89
	10%	Mean	561.07 f	488.75 e	29.01 b	3572.47 de	310.46 g	31.13 e	1095.54 bc
		SD	7.52	15.56	0.72	57.71	7.21	0.68	201.41
S. cerevisiae LOCK 0153	1%	Mean	879.85 e	1102.12 d	28.03 b	2711.79 f	623.00 e	41.00 d	1376.29 bc
		SD	7.36	11.86	1.08	15.71	3.77	0.33	39.76
	5%	Mean	808.50 e	1570.46 c	20.30 b	3724.92 d	995.98 c	105.53 a	943.35 cd
		SD	14.96	38.03	0.19	83.62	29.47	3.94	154.49
	10%	Mean	1077.41 d	1583.23 c	21.19 b	3735.67 d	849.39 d	24.73 f	1199.11 bc
		SD	17.791	23.88	0.42	62.49	24.02	1.80	202.53

nd – not detected; a-h – mean values in columns denoted by different letters differ statistically significantly (two-way ANOVA, $p < 0.05$)

Table S5. Quantitative analysis of carbonyl compounds biosynthesis in the wheat sponge fermented at 15℃. Abbreviations: "Control" indicates control sample before fermentation; "SF" indicates spontaneous fermentation.

Sample	Inoculum		Concentration [µg/kg]								
			Acetadehyde	Acetone	Isobutyraldehyde	2,3-Butanedione	Isovaleraldehyde	2-Methylbutyr-aldehyde	Hexanal	Acetaldehyde diethyl acetal	2-Pentylfuran
Control	-	Mean	nd	19.02 i	nd	nd	nd	nd	5.57 a	nd	nd
		SD	-	0.25	-	-	-	-	0.47	-	-
SF	-	Mean	nd	219.37 a	nd	57.86 a	nd	nd	nd	nd	0.08 c
		SD	-	3.01	-	1.33	-	-	-	-	0.00
S. cerevisiae D3	1%	Mean	20867.98 c	69.18 g	13.57 c	2.29 ef	7.35 b	0.42 e	1.07 c	1.08 b	0.04 f
		SD	121.28	2.54	0.06	0.09	0.03	0.02	0.14	0.08	0.00
	5%	Mean	68906.98 a	142.06 cd	41.93 a	6.65 d	11.77 a	2.11 a	2.84 b	4.17 a	0.06 e
		SD	327.66	9.40	0.16	0.79	0.19	0.01	0.05	0.06	0.00
	10%	Mean	36605.96 b	136.59 d	29.76 b	9.66 c	6.31 c	1.16 b	3.31 b	1.13 b	0.080cd
		SD	230.79	2.17	1.09	0.10	0.16	0.02	0.08	0.02	0.00
S. cerevisiae D13	1%	Mean	4762.15 g	153.38 c	4.38 f	3.00 e	nd	nd	nd	nd	0.08 cd
		SD	27.93	2.51	0.08	0.19	-	-	-	-	0.00
	5%	Mean	3519.85 h	61.76 g	9.92 d	3.11 e	2.17 f	0.26 f	nd	nd	0.07 d
		SD	14.073	0.50	0.14	0.08	0.06	0.01	-	-	0.00
	10%	Mean	5512.43 f	91.60 f	8.56 e	3.58 e	1.39 g	0.19 g	0.45 d	nd	0.09 b
		SD	61.29	0.45	0.032	0.28	0.05	0.00	0.04	-	0.00
S. cerevisiae LOCK 0153	1%	Mean	16785.00 d	104.66 e	13.98 c	5.61 d	2.43 f	0.52 d	nd	0.32 c	0.08 cd
		SD	62.44	0.71	0.14	0.36	0.03	0.01	-	0.02	0.00
	5%	Mean	15923.03 e	177.44 b	13.80 c	1.14 fg	4.11 d	1.00 c	1.46 c	0.26 c	0.12 a
		SD	178.31	2.75	0.24	0.08	0.03	0.01	0.05	0.00	0.01
	10%	Mean	1779.07 i	43.72 h	8.13 e	12.01 b	3.01 e	0.20 g	nd	nd	0.05 e
		SD	28.08	0.20	0.06	0.34	0.06	0.02	-	-	0.00

nd – not detected; a-g – mean values in columns denoted by different letters differ statistically significantly (two-way ANOVA, $p < 0.05$)

LACTIC ACID BACTERIA

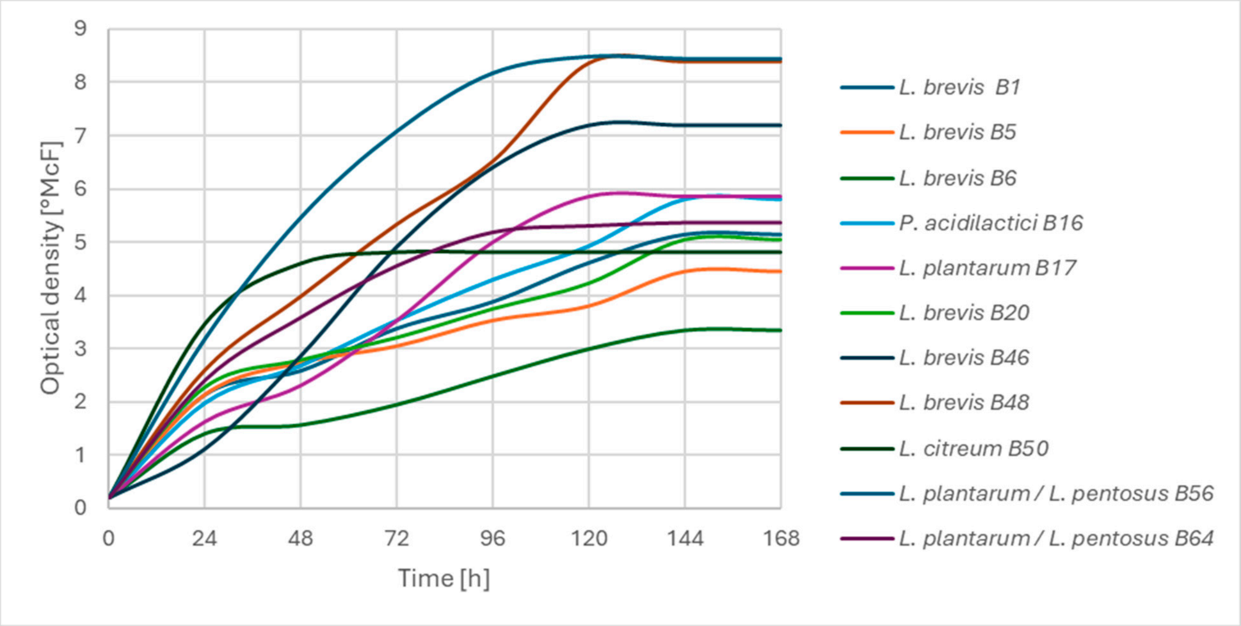

Figure S3. Growth dynamics expressed as optical density of lactic acid bacteria suspensions at 15℃.
